# From Gaze to Interaction: Links Between Visual Attention, Facial Expression Identification, and Behavior of Children Diagnosed with ASD or Typically Developing Children with an Assistance Dog

**DOI:** 10.3390/bs15050674

**Published:** 2025-05-14

**Authors:** Manon Toutain, Salomé Paris, Solyane Lefranc, Laurence Henry, Marine Grandgeorge

**Affiliations:** 1 UMR 8002 Integrative Neuroscience and Cognition Center, CNRS, Université Paris-Cité, 75006 Paris, France; 2 CNRS, EthoS (Éthologie Animale et Humaine)—UMR 6552, University Rennes, Normandie University, 35000 Rennes, France; salomeeparis@gmail.com (S.P.); solyane.lefranc@gmail.com (S.L.); laurence.henry@univ-rennes.fr (L.H.); marine.grandgeorge@univ-rennes.fr (M.G.)

**Keywords:** visual attention, assistance dog, autism, ethology, human-animal interaction

## Abstract

Understanding how children engage with others is crucial for improving social interactions, especially when one of the partners is an animal. We investigated relationships between interaction strategies, visual attention, and facial expression identification of children interacting with an assistance dog, and evaluated the effects of a neurodevelopmental disorder (Autism Spectrum Disorder (ASD)) on these elements. Thus 20 children (7 with ASD, 13 with typical development or TD) participated in three experimental tasks: (1) face-to-face encounters with the assistance dog while wearing eye-tracking glasses to analyze visual exploration patterns; (2) free interactions with the assistance dog, assessed using ethological methods and (3) a computerized task evaluating human and canine facial expression identification. The results revealed that (1) visual exploration patterns varied depending on task instructions: ASD children paid less attention to faces and more to the environment than TD children; (2) both groups displayed similar behavioral patterns during free interactions with the assistance dog; (3) facial expression identification data did not differ between groups; and (4) within-group associations emerged between visual attention, spontaneous interaction behaviors, and facial expression identification abilities. These findings highlighted the complex interplay between visual attention, facial expression identification, and social behavior of children, emphasizing the importance of context in shaping interaction strategies.

## 1. Introduction

### 1.1. Humans’ Visual Attention and Its Importance in Social Interactions

Attention is a cognitive process that allows the selection, processing, and filtering of relevant information or stimuli from the many that reach us (Posner & Rothbart, 2007). Visual attention plays a crucial role in social interactions as it enables individuals to gather information and cues from their partners, such as postures, gestures, facial expressions, or gaze direction (Langton et al., 2008). These signals help individuals infer the intentions and mental states of their communication partners (e.g., mutual gaze, joint attention; Emery, 2000), allowing them to adapt and adjust their behavior, thus facilitating smooth communication and mutual understanding (Chokron et al., 2014).

Infants exhibit early visual attention to faces, which is essential for detecting potential danger and recognizing signs of rejection, fear, acceptance, or affection (Chokron et al., 2014). This visual attention system plays a key adaptive role in ensuring the proper development of processes essential for social interactions (Nelson, 2001; Gillet & Barthélémy, 2011). Evaluation of visual attention is therefore a major methodological challenge. Data in the literature is traditionally based on visual search tasks (Gerhardstein & Rovee-Collier, 2002), target detection tasks (Johnstone et al., 2007), and reaction time tasks to visual stimuli (Posner, 1980). More recently, many studies have adopted eye-tracking techniques to record eye movements, providing a precise and non-invasive method to infer individuals’ visual attention (Valtakari et al., 2021; Thorsson et al., 2024).

### 1.2. Social Visual Attention in Autistic Spectrum Disorder (ASD)

Social visual attention of individuals with a neurodevelopmental disorder such as ASD can be different or even disrupted, leading to a so-called neuro-atypical cognitive profile. ASD is a neurodevelopmental disorder that manifests early in childhood and is characterized by a dyad of impairments: (1) deficits in communication and social interaction, including difficulties in both verbal (e.g., echolalia, language delay) and nonverbal communication (e.g., gestures, facial expressions), as well as challenges in initiating and maintaining interactions while adhering to social norms; (2) the presence of restricted and repetitive behaviors, interests and activities, including verbal and motor stereotypies (DSM-5; APA, 2013). Additionally, sensory processing difficulties are commonly observed, manifesting as hypo- or hyper-reactivity to sensory stimuli. Furthermore, the sex ratio in ASD is approximately three males for every female (Loomes et al., 2017).

Visual attention is one of the avenues explored to understand the social differences of ASD children (Courchesne et al., 1994; Chokron et al., 2014). For example, ASD children exhibit deficits in orienting their attention towards social stimuli such as faces and tend to focus more on objects compared to typically developing (TD) children (Klin et al., 2002; Riby & Hancock, 2008; Gillet & Barthélémy, 2011). Indeed, they display an atypical pattern of facial exploration, spending more time looking at a mouth and less time at the eye region compared to TD children (Klin et al., 2002; Nakano et al., 2010; Spezio et al., 2007; Grandgeorge et al., 2016; Valiyamattam et al., 2020; Dollion et al., 2021; Toutain et al., 2024). As a result, insufficient gaze toward faces and atypical exploration patterns can interfere with the processing and recognition of facial expressions. Studies show that ASD children do worse in emotion recognition tasks or facial expression identification tasks than their TD peers (Dyck et al., 2001; Bölte & Poustka, 2003; Harms et al., 2010; Black et al., 2017). Consequently, impairments in these abilities may lead to difficulties in assimilating and understanding social signals and contexts.

### 1.3. ASD Children’s Visual Attention During Interspecific Interactions

Reports increasingly stress the benefits of human-animal interactions for ASD children and a widespread approach is based on animal-assisted interventions (O’Haire et al., 2013; Davis et al., 2015). These children can be accompanied in their daily lives by several pets as well as by an assistance dog that provides multiple benefits (Dollion & Grandgeorge, 2022). This daily presence could improve their communication skills (Grandgeorge et al., 2012b; Erbacher & Bördlein, 2025). Moreover, the facial exploration strategies of ASD children improved in the presence of an assistance dog than those of ASD children living without one (Dollion et al., 2022). A recent study showed that after one school year, the presence of an assistance dog had helped adolescents with cognitive function disorders (including ASD) to identify human and dog facial expressions better (Toutain et al., 2025). To explain such benefits, the authors explore the role of visual attention. Indeed, unlike human faces, animals represent visually attractive stimuli for ASD children. These children look more at pictures of animals than at pictures of humans or objects (Celani, 2002). This preference is also observed in real-life settings with a dog (Prothmann et al., 2009; Dollion et al., 2021). These children may therefore find it easier to engage in and maintain interactions with animals rather than with other humans. More precisely, both ASD children and TD children exhibit similar visual attention patterns towards their household cats and dogs in daily life interactions (e.g., glances, gaze; Grandgeorge et al., 2020). Other reports, based on eye tracking technologies, showed that ASD children display a facial exploration pattern similar to that of TD children when looking at animal faces, with a particular interest in the eye region, that was not observed when they explore human faces (Grandgeorge et al., 2016; Valiyamattam et al., 2020; Toutain et al., 2024). This more typical exploration pattern of animal faces may enable ASD children to extract information efficiently from an animal’s face, thereby facilitating interaction regulation (Dollion et al., 2021).

Building on previous literature, we aimed to investigate the visual attention and interaction strategies of ASD children and typically developing (TD) children during real-life, unguided interactions with an assistance dog (i.e., without guidance or intervention from a referent). We also explored potential associations between their interaction strategies, visual attention patterns, and their ability to identify facial expressions. Thus our research included three sub-studies: (Study 1) characterization of facial exploration patterns (i.e., human and dog) of ASD children or TD children using a head-mounted eye-tracking approach during a real encounter; (Study 2) a behavioral analysis of these children during spontaneous interactions with the same assistance dog; (Study 3) assessment of children’s ability to identify human and canine facial expressions based on photographs. Finally, we examined the relationships between visual attention (Study 1) and (a) the interaction strategies adopted during spontaneous interactions with the assistance dog (Study 2), as well as (b) the ability to identify human and canine facial expressions (Study 3). In Study 1, we expected both groups of children (those with ASD and those with TD) to display similar visual attention patterns when observing an assistance dog, but to differ in their visual attention toward a human. In Study 2, we anticipated no significant difference between the groups in terms of spontaneous interactions with the assistance dog. In Study 3, similar to Study 1, we expected comparable performance between the groups in recognizing canine facial expressions. However, we anticipated that ASD children would perform more poorly when interpreting human facial expressions. Furthermore, we hypothesized that visual attention directed toward the assistance dog will be associated with spontaneous engagement behaviors during interactions, with longer fixation durations on the assistance dog corresponding to increased interactive behaviors. Finally, we expected visual attention toward the assistance dog to be positively correlated with performance in recognizing facial expressions, with greater attention to the assistance dog enhancing the ability to identify the emotions expressed. This study should be considered as a preliminary investigation, given its sample size limitations.

## 2. General Materials and Methods

### 2.1. Ethics

The present research was non-invasive and did not involve any pharmacological intervention. Parents provided written informed consent, and the assent of the children was obtained both orally and in writing. This study was approved by the Sud Méditerranéen III ethics committee on 5 September 2022, [N°2022.06.09 bis_22.02106.000105].

Regarding ethical considerations for the assistance dogs, the experiments were conducted in accordance with Directive 2010/63/EU of the European Parliament and of the Council on the protection of animals used for scientific purposes. They complied with current French legislation on animal experimentation (Decree No. 2013-118 of 1 February 2013) and its five implementing orders (JO 7 February 2013), integrated into the Rural and Maritime Fishing Code (Articles R.214-87 to R.214-137). The procedures fell outside the scope of the European Directive, as they involved only behavioral observations and non-invasive interactions. In accordance with French regulations, no ethical authorization was required. The assistance dogs involved were not research animals; their care and housing were entirely managed by the professional trainers of the Handi’Chiens association. They were accustomed to human interaction, and each participated in a maximum of five sessions per day, with a minimum of 30 min of rest between each 10-min session. All sessions took place on the association’s premises, in a familiar environment located approximately 100 m from the assistance dogs’ living area, eliminating the need for transport.

### 2.2. Participants

Humans. The inclusion criteria in our study were being between 6 and 17 years old and having ASD or typical development. The exclusion criteria were having epilepsy, having a significant intellectual disability making it too difficult to understand our instructions, or requiring strong visual correction (an informal visual acuity test was conducted beforehand by the experimenter). Thus, twenty French children (11 girls and 9 boys) between 6 and 16 years old (mean ± SD: 9.7 ± 2.6 years old) participated in this study. Seven of them were diagnosed with ASD (7 boys; 10.6 ± 3.0 years old), and thirteen had typical development (11 girls and 2 boys; 9.3 ± 2.0 years old) (for details, see Table 1). Ages did not differ significantly between the two groups (Mann-Whitney test, W = 55.5, *p* = 0.4452). In the informational questionnaire completed by the parents, only one parent of a typically developing child mentioned that their child could be afraid of unfamiliar dogs.

The parents of all the children fulfilled two questionnaires. First, the Social Communication Questionnaire (SCQ, a 40-item parent-report screening questionnaire for ASD, Rutter et al., 2003). We used the SCQ French version (Kruck et al., 2013). Items on the SCQ correspond to criteria used to diagnose the core features of ASD (i.e., communication, reciprocal social interactions, and repetitive behaviors and interests). This autism screening questionnaire is the best researched and validated parent-report screening tool for ASD (Norris & Lecavalier, 2010) as it shows strong discrimination between ASD and non-ASD cases as well as good reliability and validity (Witwer & Lecavalier, 2007). The lifetime version was used in our research. Each item on the SCQ is rated as ‘‘yes’’ or ‘‘no’’ and assigned a 0–1 point rating (0 = absence of abnormal behavior, 1 = presence of abnormal behavior). Items address both current and past behavior. The possible range of scores for nonverbal children is 0–33 and for verbal children is 0–39. In the French version, the cut-off score used for ASD screening purposes is 15 (Kruck et al., 2013). Second, we also used the short version of the Dunn Sensory Profile (Dunn, 2010) designed for 3 to 10-year-old and 11-month-old children. This questionnaire assesses a child’s sensory sensitivity. A score between 38 and 122 indicates a significant sensory difference compared to a group of typically developing (TD) children. A score between 123 and 149 suggests a probable sensory difference, while a score between 150 and 190 corresponds to typical performance.

Dogs. Five assistance dogs (all Golden Retrievers, 1 female, and 4 males; 3.2 ± 2.1 years old), trained by the Handi’chiens association, participated in the study. Each child was randomly assigned an assistance dog.

### 2.3. Study Context

The study took place at the Handi’Chiens training center in Alençon, France, within the “Autism” building. A dedicated room was provided for the study, equipped with a desk, chairs, a sofa, a bed, a carpet, and a coffee table with objects belonging to the assistance dog (i.e., a knotted rope, an intelligence toy for dogs (plastic platform with compartments to hide treats, specially designed for dogs), a toy veterinary kit (composed entirely of wooden objects mimicking real medical instruments used for care, such as a toy stethoscope and a toy syringe, all neatly packaged in a small fabric bag), and a brush). Additionally, a watering can be filled with water and a food bowl was placed near the bed. A parent was present throughout the session but remained non-intervening. The room was equipped with three Sony Camcorder FDR-AX43A Black, Full HD cameras.

### 2.4. Methodology

Each participant completed the three studies consecutively, always in the same order, during a single appointment. Details of the studies are given below.

### 2.5. Statistical Analyses

All statistical analyses were conducted using RStudio software version 2023.09.0. The significance threshold was set at 0.05, and non-parametric statistics were used. Detailed information about statistical analyses is provided in each of the following subparts.

## 3. Study 1: Characterization of ASD Children and TD Children’s Visual Exploration Pattern

### 3.1. Methods

#### 3.1.1. Participants

Seventeen of the twenty children in the research participated in this study (9 girls and 8 boys). Among them, 6 boys were diagnosed with ASD and 11 had typical development (9 girls and 2 boys). Their mean age was 10.2 ± 2.6 years. Ages did not differ significantly between the two groups (Mann-Whitney test, W = 41, *p* = 0.4419).

#### 3.1.2. Procedure

Eye fixations were collected and recorded using Tobii Glasses Pro 2 eye-tracking glasses (Appendix A). The glasses were connected to a power unit, which transmitted real-time data to a computer. Data recording was performed using the Tobii Pro Glasses Controller software (version 1.1 4.20033). The procedure followed a standardized scenario, as described below. The child sat on a carpet on the floor. The experimenter fitted the glasses and performed a single-point calibration. Eye-tracking measures are detailed in Table 2 (total average data collection time: mean ± SD: 26.1 ± 5.1 s).

*Phase 1. Assistance dog presentation (8.3 ± 1.1 s)*^1^. The experimenter entered the room with an assistance dog on a leash and instructed the dog to lie down or sit. Then, she introduced the dog, providing its name, age, breed, and color. Eye-tracking coding started when the experimenter said “Let me introduce you to” and ended 20 s later.

*Phase 2. Questions about assistance dogs (9.1 ± 2.3 s)*. The experimenter asked the child two questions about the assistance dog: “What do you think of it?” and “How do you think it feels?” Eye-tracking coding started when the first question was asked and ended after the child’s final response.

*Phase 3. Gaze fixation on the experimenter (4.1 ± 1.2 s) and the assistance dog (4.6 ± 0.5 s).* The experimenter asked the child to fixate on her for 10 s and then to fixate on the dog for another 10 s. Only the first 5 s were coded. This duration was chosen based on previous eye-tracking studies using similar protocols for animal face presentation on a screen, typically lasting 5 s (Muszkat et al., 2015; Grandgeorge et al., 2016; Valiyamattam et al., 2020). For both fixation tasks, eye-tracking coding started when the experimenter began counting aloud from “1” and ended before the number “6” was spoken. Given the time-consuming nature of manual coding and the standard presentation times observed in eye-tracking studies on screens (typically between 3 and 5 s), this duration was deemed appropriate.

#### 3.1.3. Data Collection and Statistical Analyses

The collected data corresponded to the fixation time on different areas of interest (AOIs) of an assistance dog’s face (i.e., AOIs: head, eyes, muzzle, ears) and the experimenter’s face (i.e., AOIs: head, eyes, nose-mouth), as well as “other” AOI encompassing the entire visual scene (Table 2). AOIs were manually adjusted to accommodate changes in the configuration of the faces in the scene (e.g., when an assistance dog was in profile). Since the amount of usable data varied between participants, fixation times were converted into percentages to allow comparisons.

Mean comparison tests (i.e., Friedman test, Wilcoxon test with “FDR” correction, and Mann-Whitney test) compared facial exploration pattern data between the two groups of children and within each group.

### 3.2. Results

#### 3.2.1. Visual Exploration of ASD Children

**Figure 1:**
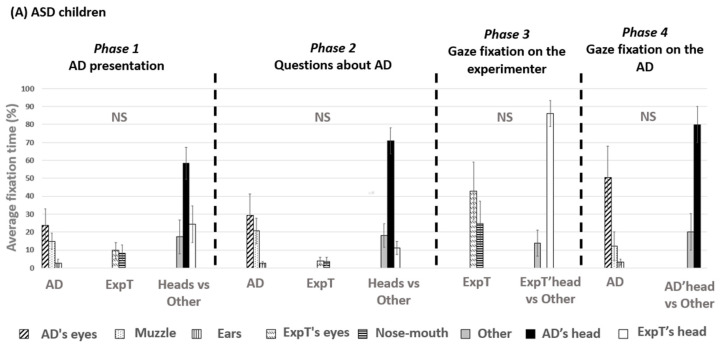

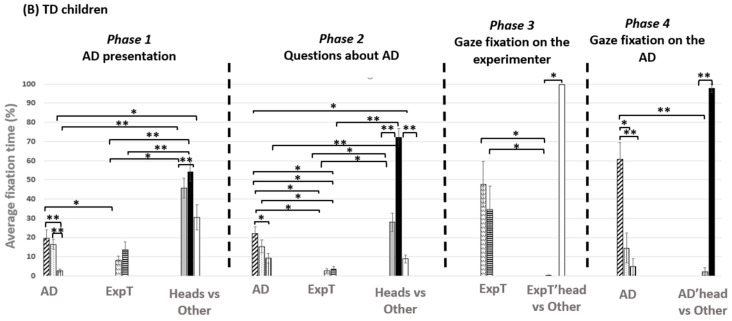
Fixation times (mean ± S.E.) for the different Areas Of Interest (AOIs) during the different phases (**A**) for ASD children and (**B**) and TD children. AD: Assistance Dog, ExpT: Experimenter. *: *p* < 0.05, **: *p* < 0.01, NS: Non-significant. Wilcoxon tests.

The data are represented in Figure 1A (see Appendix B for details in percent).

*Phase 1. Presentation of an assistance dog.* ASD children did not seem to spend the same amount of time on each AOI (X^2^ = 18.7; df = 7; *p* = 0.009). However, pairwise comparisons could not reveal any significant differences between AOIs (all *p* > 0.05). Nevertheless, the longest fixation time percentage (58.32 ± 21.75%) was for an assistance dog’s head, while the shortest was for a dog’s ears (2.75 ± 5.77%).

*Phase 2. Questions about the assistance dog.* ASD children did not seem to spend the same amount of time on each AOI (X^2^ = 22.7; df = 7; *p* = 0.002). However, pairwise comparisons could not reveal any significant differences between AOIs (all *p* > 0.05). During this phase, the fixation time percentage was the highest for an assistance dog’s head (70.80 ± 17.75%), and the lowest percentage was for an assistance dog’s ears (2.71 ± 1.84%).

*Phase 3. Gaze fixation on the experimenter.* ASD children did not seem to spend the same amount of time on each AOI (X^2^ = 9.9; df = 3; *p* = 0.019). However, pairwise comparisons could not reveal any significant differences between AOIs (all *p* > 0.05). During this phase, the highest fixation time percentage was for the experimenter’s head (86.0 ± 17.78%), and the lowest percentage was for AOI “other” (14.0 ± 17.78%).

*Phase 4. Gaze fixation on an assistance dog.* ASD children did not seem to spend the same amount of time on each AOI (X^2^ = 14.6; df = 5; *p* = 0.006). However, pairwise comparisons could not reveal any significant differences between AOIs (all *p* > 0.05). During this phase, the highest fixation time percentage was for a dog’s head (79.95 ± 24.81%), and the lowest percentage was for AOI “other” (20.05 ± 24.81%).

#### 3.2.2. Visual Exploration of TD Children

The corresponding data are represented in Figure 1B (see Appendix B for percentage details).

*Phase 1. Assistance dog presentation.* TD children did not spend the same amount of time on each AOI (X^2^ = 45.3; df = 7; *p* < 0.001). They gazed more at an assistance dog’s head than at the “other” AOI, the experimenter’s eyes, and the experimenter’s nose-mouth region (respectively, W = 2; *p* = 0.008; W = 66; *p* = 0.003; W = 66; *p* = 0.003). They gazed less at an assistance dog’s ears than at the human’s head, the assistance dog’s head, the assistance dog’s muzzle, and the “other” AOI (respectively: W = 63; *p* = 0.01; for the other three: W = 66; *p* = 0.003). They gazed less at the experimenter’s eyes than at the “other” AOI and the assistance dog’s eyes (for both: W = 61; *p* = 0.02).

*Phase 2. Questions about the assistance dog.* TD children did not spend the same amount of time on each AOI (X^2^ = 41.6; df = 7; *p* < 0.001). They gazed more at an assistance dog’s head than at the following AOIs: the “other” AOI, the human’s head, the human’s eyes, and the human’s mouth (respectively: W = 0; *p* = 0.008; for the next three: W = 55; *p* = 0.008). Then, they gazed more at an assistance dog’s eyes than at the human’s head, nose-mouth region, eyes, and the assistance dog’s ears (respectively: W = 49; *p* = 0.04; W = 54; *p* = 0.01; W = 54; *p* = 0.01; W = 51; *p* = 0.02). They gazed more at an assistance dog’s muzzle than at the human’s nose-mouth region and eyes (respectively: W = 54; *p* = 0.01; W = 52; *p* = 0.02). Finally, they gazed more at “other” AOI than at the human’s nose-mouth region and eyes (respectively: W = 50; *p* = 0.03; W = 53; *p* = 0.01).

*Phase 3. Gaze fixation on the experimenter.* TD children did not spend the same amount of time on each AOI (X^2^ = 23.5; df = 3; *p* < 0.001). They gazed more at the experimenter’s head than at the “other” AOI (W = 0; *p* = 0.01). They gazed more at the experimenter’s eyes and nose-mouth AOI than at the “other” AOI (W = 0; *p* = 0.02; W = 0; *p* = 0.04). No significant differences were evidenced between fixation times for the eyes and the nose-mouth AOIs (*p* > 0.05).

*Phase 4. Gaze fixation on the assistance dog.* TD children did not spend the same amount of time on each AOI (X^2^ = 36.4; df = 4; *p* < 0.001). They spent more time looking at a dog’s head than at the “other” AOI (W = 0; *p* = 0.005). They looked more at a dog’s eyes than at its muzzle, ears, and the “other” AOI (W = 9; *p* = 0.04; W = 64; *p* = 0.005; W = 0; *p* = 0.003).

### 3.3. Comparisons Between ASD Children and TD Children

No significant differences could be evidenced for gaze times for the different AOIs between the two groups during the presentation phase (Phase 1) or the question phase (Phase 2) (all *p* > 0.05).

**Figure 2:**
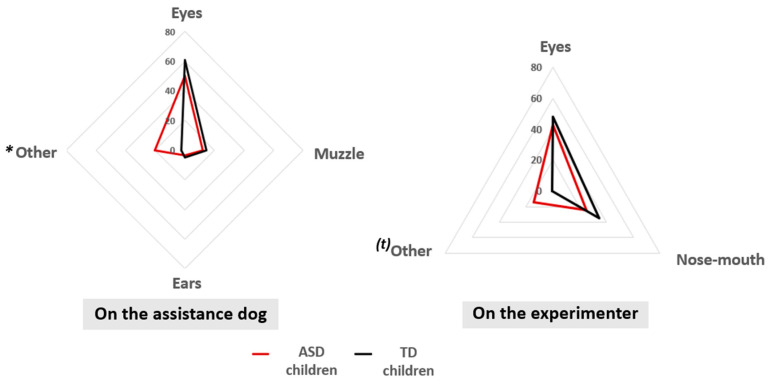
Fixation times (mean) on different areas during the fixation phases on the assistance dog (**left**) and the experimenter (**right**) for ASD children (red) and TD children (black). *: *p* < 0.05, (*t*): *p* < 0.10. Mann-Whitney test.

During the experimenter fixation phase (Phase 3), ASD children tended to fixate more on the AOI “other” than did TD children (W = 43.5; *p* = 0.064) (Figure 2). ASD children tended to look less at the experimenter’s face than did TD children (W = 16.5; *p* = 0.064). During the assistance dog fixation phase (Phase 4), TD children looked more at a dog’s face than ASD children (W = 15; *p* = 0.039). ASD children looked more at the “other” AOI than did TD children (W = 51; *p* = 0.039).

## 4. Study 2: Spontaneous Interactions Between Children and Assistance Dogs

### 4.1. Participants

All the children participated in this study (*n* = 20). For a detailed description of the population, see Table 1. Ages did not differ significantly between the two groups (Mann-Whitney test, W = 55.5, *p* = 0.4452).

### 4.2. Procedure

The protocol for Study 2 followed a standardized scenario, described below (Table 2). Only one parent was present in the room, seated in the same location against a wall. They were instructed to not interfere in the interactions between the child and the dog, except in a dangerous situation requiring intervention (which did not occur). If a child sought their attention, they were allowed to respond.

*Phase 1. Explanation (2 to 5 min).* The experimenter presented various objects (i.e., the puzzle game, the toy veterinary kit, the brush, the knotted rope, the watering can, and the water bowl) to the child and explained what the child could do with each one. The experimenter then left the room, brought the assistance dog in on a leash, and then released the assistance dog. She placed the leash with the other dog’s objects and told the child she would return in 10 min. The time was indicated by a timer visible to the child.

*Phase 2. Spontaneous interaction (10 min 28 ± 27 s)*. The child interacted freely with the assistance dog for 10 min. The behavioral coding phase began when the experimenter left the room, closed the door, and the door handle no longer moved. It ended when the timer went off, signaling the end of the 10 min.

Note that throughout the spontaneous interaction phase, the experimenter was in the next room, monitoring the interactions between the child and the assistance dog through a one-way mirror. If necessary, the experimenter could intervene at any time if either the child or the assistance dog was in difficulty. However, this never occurred during our study.

### 4.3. Data Collection and Statistical Analyses

The videos were converted using the ShotCut application and then coded with The Observer software (Version XT 16). The behaviors recorded are described in Table 3. Scan sampling (Altmann, 1974) was used at a rate of one scan every 5 s to collect distances, the presence or absence of play, and a child’s visual attention. Focus sampling (Altmann, 1974) was used to record the occurrences of contacts, locomotor behaviors, care behaviors, verbalizations, onomatopoeias, laughter and smiles, joint attention, self-centered behaviors, and finally, verbal and motor stereotypes. Durations were only collected for physical contact.

Our statistical analyses involved the fact that the total durations of the videos as well as the durations and occurrences of the variables were standardized to 10 min (range: 9 min 0 s to 10 min 6 s). Mean comparison tests were performed using Friedman tests, Wilcoxon tests with “FDR” correction, and Mann-Whitney tests.

### 4.4. Results

#### General Description for ASD Children and TD Children

*Time budget for interpersonal distances between children and an assistance dog (Table 4).*

Children did not spend the same amount of time at different distances from the assistance dog (ASD: Χ^2^ = 19.8, df = 4, *p* < 0.001; TD: Χ^2^ = 42.9, df = 4, *p* < 0.001). TD children interacted significantly more often at less than one arm’s length from the assistance dog than at 1–2 arms (W = 91, *p* < 0.001) or beyond 2 arms (W = 91, *p* < 0.001). Physical contact with an assistance dog was significantly more frequent than interactions at 1–2 arms (W = 81, *p* = 0.013) or beyond 2 arms (W = 87, *p* = 0.002). In contrast, the positions of ASD children in relation to dogs were less pronounced. The contacts of these children with an assistance dog did not differ significantly in relation to distance (less than one arm, 1–2 arms, and more than 2 arms, *p* > 0.05). However, contacts with the assistance dog were more frequent than interactions at 1–2 arms (W = 28, *p* = 0.031), and the distance of less than one arm was observed more frequently than beyond 2 arms (W = 28, *p* = 0.031).

*Time budgets of interpersonal distances between children and their parents (* Table 4 *).*

Distances between a child and his/her parent varied significantly (ASD: X^2^ = 22.8; df = 4; *p* < 0.001; TD: X^2^ = 43.8; df = 4; *p* < 0.001). However, ASD children tended to be farther from the parent (i.e., more than 2 arms versus contact, less than one arm, and between 1 and 2 arms; for all, W = 0, *p* = 0.055). TD children were significantly more often observed at a distance of 2 arms from the parent compared to contact (W = 0, *p* = 0.004), less than one arm (W = 0, *p* = 0.004), and between one and two arms (W = 0, *p* = 0.013). Additionally, TD children spent less time at a distance of less than one arm from their parent than at a distance of one to two arms (W = 0, *p* = 0.013).

No significant differences could be evidenced between the two groups regarding distances from the dog or parent (all *p* > 0.05).

*Time budgets for activities (play/non-play) with an assistance dog (*Table 5*).* Children did not spend the same amounts of time in different activities with a dog (ASD: X^2^ = 17.7; df = 5; *p* = 0.003; TD: X^2^ = 37; df = 5; *p* < 0.001). TD children spent more time “non-playing” than playing, whatever the type of play (W = 0, *p* < 0.001). Additionally, TD children played significantly more with the rope than played with the toy veterinary kit (W = 10, *p* = 0.048). No other significant differences could be evidenced (all *p* > 0.05). No significant differences could be evidenced between ASD children’s different play activities (all *p* > 0.05). Finally, intergroup comparisons (ASD vs. TD) could not reveal any differences between types of play (all *p* > 0.05).

*Time budgets of children’s visual attention (*Table 6*).* Children did not spend the same amounts of time observing the different targets (ASD: X^2^ = 26; df = 5; *p* = 0.003; TD: X^2^ = 54.7; df = 5; *p* < 0.001). TD children spent more time looking at their assistance dog than at their parent (W = 91, *p* < 0.001), an assistance dog’s objects (W = 91, *p* < 0.001), or the environment (W = 91, *p* < 0.001). ASD children spent more time looking at an assistance dog than at their parent (W = 28, *p* = 0.033) and tended to look at the assistance dog’s objects more than at the other targets (W = 27, *p* = 0.052). Intergroup comparisons revealed that TD children tended to look at the assistance dog more than ASD children (W = 20.5; *p* = 0.052).

*Nature of contact with an assistance dog (*Table 7*).* TD children’s active contacts with a dog were longer than their passive contacts (W = 82; *p* = 0.008). Additionally, they made active contact more frequently than passive contact (W = 85, *p* = 0.007). In contrast, no such differences could be evidenced for ASD children (occurrence and duration; *p* > 0.05). Similarly, the intergroup comparison did not reveal any significant differences concerning the type of contact (all *p* > 0.05).

*Vocalizations directed to a dog and parent (*Table 8*).* Type of vocalizations to a dog varied (ASD: X^2^ = 9.55, df = 2, *p* = 0.008; TD: X^2^ = 19.86, df = 2, *p* < 0.001). ASD children tended to encourage interactions vocally more than interrupt them (W = 21, *p* = 0.054). ASD children also emitted more neutral vocalizations directed to a dog aimed at interrupting an interaction (W = 28, *p* = 0.047). Types of vocalization directed to the parent by ASD children did not differ significantly (*p* > 0.05). TD children emitted vocalizations that encouraged interaction with a dog more than vocalizations aimed at interrupting an interaction (W = 91, *p* = 0.0004). Similarly, TD children emitted more neutral vocalizations towards a dog than vocalizations aimed at stopping an interaction (W = 91, *p* = 0.0004). TD children were more likely to talk about the dog with their parents than to talk about other things (W = 68, *p* = 0.025). Intergroup comparisons could not reveal any significant differences regarding the different vocalizations (all *p* > 0.05).

## 5. Study 3: Facial Expression Identification Skills

### 5.1. Participants

All children participated (*n* = 20) in this study. For the description of the population see Table 1. Ages did not differ significantly between the two groups (Mann-Whitney test, W = 55.5, *p* = 0.4452).

### 5.2. Procedure

The protocol for study 3 followed a previously used standardized scenario (Toutain et al., 2025) based on the presentation of visual stimuli on a 13-inch computer screen (Dell, Latitude 5420).

*Phase 1. Definition of facial expressions*. Before starting, a child was asked to define five facial expressions: sadness, joy, fear, anger, and neutral, and then the experimenter provided a definition without giving any configurational details.

*Phase 2. Instructions given to child.* Once a child was seated in front of the computer, the experimenter gave the instructions: “I will now show you a series of photos of human and animal faces. For each face, you must tell me which emotion^2^ best matches the face you see. To help you, I’ve placed the names of the possible emotions in front of you.” Five rectangular labels with the five facial expressions written on them were placed in front of the child. The experimenter reassured the child by explaining that there were no right or wrong answers and that only their opinion mattered.

*Phase 3. Testing phase*. The stimuli were presented in blocks, with each block corresponding to a species: either human or dog. There were two pictures per facial expression of each species, meaning there were 10 faces per block. The two blocks were presented consecutively in a random order. The subject had to name the facial expression on each of the 20 stimuli, either by saying it aloud or by pointing to the corresponding label. This task was not subject to a time constraint.

### 5.3. Visual Stimuli

Two categories of photographs were used: dog faces and human faces. The dog photographs included five images of the same golden retriever (the same breed as the assistance dog), each showing a different facial expression (i.e., sadness, joy, fear, neutral, anger), and five images of different dogs of various breeds, each showing a different facial expression. The human photographs included five women and five men, also showing five different facial expressions. The photographs of dogs were sourced from Bloom and Friedman (2013); Borgi et al. (2014), Inès Sauvage, Pxhere, Kaggle, and authors’ personal sources. The photographs of humans were extracted from the FACES databases. These pictures were previously used for a similar protocol (Toutain et al., 2025), although here, we focused only on dog and human pictures. Thus, our full test set consisted of 20 photographs of two species, each showing five different facial expressions (i.e., sadness, joy, fear, neutral, anger), with two examples of each expression. The backgrounds of all the photographs were standardized: the background was removed from all the dog photographs and all the human photographs a given a uniform light grey background. All photographs showed individuals facing the camera. The dog photographs measured 10.3 ± 1 × 9.9 ± 0.67 cm, and the human photographs measured 19 × 15.2 cm.

### 5.4. Data Collection and Statistical Analyses

The experimenter recorded immediately the subject’s responses for each stimulus on paper, and these responses were later scored on a binary scale (i.e., 1 = success and 0 = failure). For this study, multivariate analyses were conducted using binomial Generalized Linear Mixed Models (GLMMs) built using the GLMER function in R (“lme4” package), followed by a type II ANOVA (“Anova” function in R -“car” package) on the models to assess the influence of various factors on children’s ability to identify facial expressions. To assess differences between the levels of a variable (e.g., facial expression) in the model, we computed a post hoc analysis using the “glht” function in R using the Tukey method. Additionally, Mann-Whitney mean comparison tests compared the two groups of children.

### 5.5. Results

#### 5.5.1. Both Groups of Children

The diagnosis did not influence success rates significantly in the identification of facial expressions of dog or human stimuli, nor overall results (all *p* > 0.05).

*General results.* The GLMM model Result ~ Diagnosis + Facial Expression + Species + Age + (1|Individual) was used. The results were influenced by facial expression (X^2^ = 27.7; df = 4; *p* < 0.001), species (human, dog) (X^2^ = 43.3; df = 1; *p* < 0.001), and child’s age (X^2^ = 26.3; df = 7; *p* < 0.001). Older children performed better than younger ones. Human facial expressions were identified better than dog facial expressions (Z = 6.5; *p* < 0.001). Children identified anger better than fear (Z = −4.2; *p* < 0.001), sadness (Z = −4.7; *p* < 0.001), and neutral expression (Z = −3.5; *p* = 0.003). They identified joy better than sadness (Z = −3.2; *p* = 0.01).

**Figure 3:**
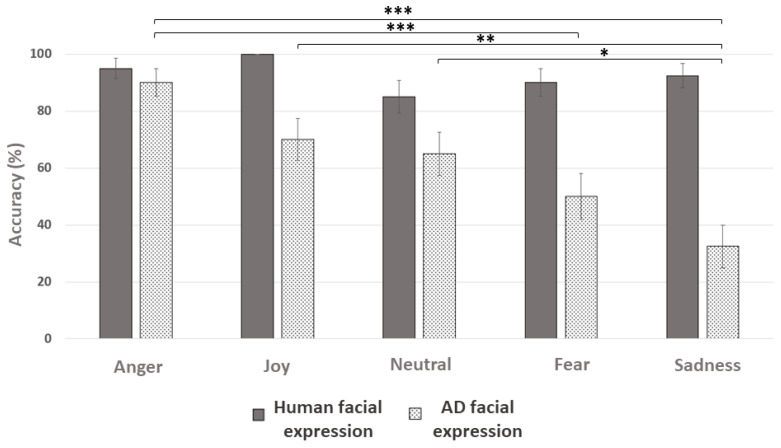
Success rates (mean percentage) of all children for human (grey) and canine (dashed) facial expressions, AD: Assistance Dog. *: *p* < 0.05, **: *p* < 0.01, ***: *p* < 0.001. Post hoc analysis using the “glht” function in R with the Tukey method.

*Results for dog stimuli (*Figure 3*).* The GLMM model ResultDog ~ Diagnostic + Facial Expression + Age + (1|Individual) was used. Facial expression (X^2^ = 28.9; df = 4; *p* < 0.001) and age (X^2^ = 22.2; df = 7; *p* = 0.002) influenced facial expression identification results for dogs. Again, older children had better results than younger ones (X^2^ = 22.2; df = 7; *p* = 0.002). They tended to identify anger better than the neutral expression (Z = −2.7; *p* = 0.053) and identified significantly better anger than sadness (Z = −4.9; *p* < 0.001) and fear (Z = −3.8; *p* = 0.001). They identified joy better than sadness (Z = −3.4; *p* = 0.005), and the neutral expression better than sadness (Z = −3.05; *p* = 0.02).

*Results for human stimuli (*Figure 3*).* The GLMM model ResultHuman ~ Diagnostic + Facial Expression + Age + (1|Individual) was used. No variable significantly influenced the facial expression identification results for the human species (*p* > 0.05).

#### 5.5.2. ASD Children

*General results.* The GLMM model ResultASD ~ Facial Expression + Species + Age + (1|Individual) was used. Species (X^2^ = 17; df = 1; *p* < 0.001) and child’s age (X^2^ = 17.3; df = 5; *p* = 0.004) influenced their overall results. ASD children identified human facial expressions better than dog facial expressions (Z = 4.1; *p* < 0.001), and older children also obtained better results.

*Results for dog stimuli.* The GLMM model ResultASDDog ~ Facial Expression + Age + (1|Individual) was used. Facial expression tended to influence (X^2^ = 8.5; df = 4; *p* = 0.07), and age significantly influenced (X^2^ = 15.4; df = 5; *p* = 0.008) their results. Older children performed better than younger ones (X^2^ = 15.38; df = 5; *p* = 0.008), and children identified anger better than sadness (Z = −2.8; *p* = 0.032).

*Results for human stimuli*. The GLMM model ResultASDHuman ~ Facial Expression + Age + (1|Individual) was used. No significant variables were found (all *p* > 0.05).

#### 5.5.3. TD Children

*General results.* The GLMM model ResultTD ~ Facial Expression + Species + Age + (1|Individual) was used. Species (X^2^ = 27.5; df = 1; *p* < 0.001) and facial expression (X^2^ = 25.8; df = 4; *p* < 0.001) influenced their results, as well as age (X^2^ = 16.7; df = 6; *p* = 0.01). TD children identified human facial expressions better than dog expressions (Z = 5.2; *p* < 0.001). They identified anger better than fear (Z = −4; *p* < 0.001), sadness (Z = −4.2; *p* < 0.001), and the neutral expression (Z = −2.9; *p* = 0.027). They identified joy better than fear (Z = −3; *p* = 0.021) and sadness (Z = −3.1; *p* = 0.01). Younger children obtained poorer results than older ones.

*Results for dog stimuli.* The GLMM model ResultTDDog ~ Facial Expression + Age + (1|Individual) was used. Facial expression influenced their results (X^2^ = 22.6; df = 4; *p* < 0.001). TD children identified anger better than fear (Z = −3.4; *p* = 0.005) and sadness (Z = −3.9; *p* < 0.001). They identified joy better than sadness (Z = −3.3; *p* = 0.009) and the neutral expression better than sadness (Z = −2.8; *p* = 0.043).

*Results for human stimuli.* The GLMM model ResultTDHuman ~ Facial Expression + Age + (1|Individual) was used. No significant variables were found (all *p* > 0.05).

## 6. Interactions Between the Results of Study 1 and 2, and Study 1 and 3

To explore the relationships between the different studies, correlation tests (i.e., Spearman’s test) were conducted.

### 6.1. Correlations Between Visual Attention and Spontaneous Interaction

*ASD children, for all phases*. For all phases of study 1, the average duration of fixation on the assistance dog’s head was correlated positively with time spent looking at the dog during an interaction (R = 0.94; *p* = 0.005), time spent in contact with the dog (R = 0.83; *p* = 0.042), duration of passive contacts (R = 0.83; *p* = 0.042), and playing with the intelligence toy (R = 0.93; *p* = 0.005). Negative correlations were also found between the average fixation duration of the AOI “other” and time spent looking at the dog during an interaction, time spent in contact with it, and duration of passive contacts (for all three variables: R = −0.83; *p* = 0.042). Average fixation time on a dog’s head was correlated negatively with the number of vocalizations to their parent about something other than the dog (R = −0.83; *p* = 0.042) and time spent within 1 to 2 arms of their parent (R = 0.83; *p* = 0.042).

*TD Children for all phases.* For all phases of study 1, average fixation duration on a dog’s head was correlated positively with time spent playing with the rope (R = 0.63; *p* = 0.039) but correlated negatively with time spent looking at their parent (R = −0.64; *p* = 0.033). Average fixation duration on a dog’s eyes was correlated negatively with time spent more than two arms away from the dog (R = −0.65; *p* = 0.032).

### 6.2. Correlations Between Visual Attention and Facial Expression Identification Ability

*ASD children for all phases.* For all phases of study 1, a positive correlation was found between average fixation duration on a dog’s eyes and facial expression identification scores for dog stimuli (R = 0.93; *p* = 0.008), human stimuli (R = 0.88; *p* = 0.021), and overall scores (R = 0.93; *p* = 0.008). Conversely, average fixation duration on a dog’s snout was correlated negatively with facial expression identification scores for dog stimuli (R = −0.9; *p* = 0.015) and overall scores (R = −0.9; *p* = 0.015). Similarly, average fixation duration on the AOI “other” was correlated negatively with facial expression identification scores for dog stimuli (R = −0.81; *p* = 0.049), human stimuli (R = −0.88; *p* = 0.021), and overall scores (R = −0.81; *p* = 0.049).

*TD Children for all phases.* For all phases of study 1, the average fixation duration on a dog’s eyes was correlated positively with facial expression identification scores for dog stimuli (R = 0.64; *p* = 0.034). Average fixation duration on a dog’s head was correlated positively with overall facial expression identification scores (R = 0.61; *p* = 0.046) and identification scores for human stimuli (R = 0.77; *p* = 0.006). Average fixation duration on the experimenter’s eyes tended to be correlated negatively with success in identifying human facial expressions (R = −0.6; *p* = 0.052).

## 7. Discussion

Our research is based on the characterization of visual attention, facial expression identification, and behaviors of ASD children or typical development with an assistance dog, and the identification of possible links between these factors. Through the integration of findings from three studies, we could highlight several key findings: (1) context influenced visual patterns, differences depending on instructions, particularly for children with typical development (TD), and divergences occurred between groups when the subjects were explicitly asked to look at their interaction partner (i.e., human or assistance dog). ASD children paid less attention to faces and focused more on the environment; (2) behaviors of the two groups observed during spontaneous interaction with the assistance dog were similar; (3) performances for the facial expression identification tasks were comparable between groups; as well as (4) within each group, links were established between visual attention, certain spontaneous behaviors during interactions, and their ability to identify facial expressions.

### 7.1. Convergences in Interaction with Assistance Dog

During interactions with an assistance dog, all children, both ASD and TD, displayed similar behaviors. However, visual attention varied during spontaneous interactions: whereas a dog was the preferred target for all children, ASD children looked at the dog less than TD children. Additionally, TD children looked at the dog more than at any other target (i.e., environment, dog-related objects, parent), whereas ASD children only looked at the dog more than at their parent.

During the eye-tracking phases when they were instructed to fixate on the experimenter and then the dog, ASD children observed the dog’s head less and focused more on the environment, unlike TD children, who fixated more on faces. These results align with previous studies showing that ASD individuals focus less on human faces (Klin et al., 2002; Riby & Hancock, 2008; Jones & Klin, 2013; Vacas et al., 2021) and animal faces (Grandgeorge et al., 2016; Valiyamattam et al., 2020) than do TD individuals (i.e., control group). However, our study introduces gradation, as no such differences were found during the question and presentation phases. This suggests that these attentional differences may be exacerbated by the explicit instruction to look at interaction partners, whatever their species. Similar results were reported in a study using remote eye tracking during social interactions, divided into different contexts, involving adults with autistic traits (Thorsson et al., 2024). In situations where they were engaged in listening and guessing activities, the expression of their autistic traits was less pronounced regarding eye contact with the experimenter. This suggests that the interaction context, characterized by active engagement and the need to interpret verbal cues, might mitigate the effects of autistic traits on visual behaviors. However, when the task involved word description, participants tended to reduce their eye contact, which could be interpreted as a response to social pressure or cognitive overload related to expressing their own thoughts and feelings (Thorsson et al., 2024). Participants who experienced discomfort with eye contact exhibited a significant reduction in their gaze at the experimenter’s eyes, indicating that the emotional and physical context of the interaction can modulate visual engagement ability. More broadly, context is crucial when evaluating behavioral expression related to ASD. For example, the imitation abilities of ASD individuals depend on the experimental context (Ingersoll, 2008), and their empathy capacity differs according to the emotional context (Hudry & Slaughter, 2009).

The different components of the interactions with assistance dogs measured here suggest convergences between the two groups of children. Firstly, both ASD children and TD children primarily interacted in close proximity to the dog while maintaining a greater distance from their parents. This finding aligns with Dollion et al. (2021, 2022), which identified different interaction profiles of ASD children with an assistance dog. Specifically, three distinct profiles have been described: the proximal-to-the-dog profile, the distal-to-the-dog profile, and the profile dependent on parental supervision (Dollion et al., 2021). The existence of different interaction profiles with an unfamiliar animal has also been documented in the presence of a guinea pig (Grandgeorge et al., 2012a). In that study, ASD children were categorized into three groups: those attracted to the animal (“confident” profile), those engaging more with their parent despite their non-participation (“human-oriented” profile), and those who showed little interest in either the animal or the parent (“self-centered” profile). We did not observe the parental supervision profile in the present study, probably due to the instructions given to parents to refrain from intervening in the interactions between the child and the dog. Increasing the sample size would be valuable for exploring whether additional interaction profiles would emerge for the distal-to-the-dog profile, as observed for interactions with a guinea pig. Indeed, this unsupervised interaction-with-dogs context has not yet been documented in the literature, particularly in comparison with TD children. The TD children in our study also interacted in close proximity to the dog. This aligns with previous research indicating that physical contact between young children (under five years old) and their pet dogs is frequent (Filiâtre et al., 1986; Millot & Filiâtre, 1986). Given that tactile contact is a preferred modality of interaction between children and animals (Montagner, 2002), we can hypothesize that similar behaviors could be observed in older children. However, to our knowledge, no study has precisely reported interaction distances between typically developing children and an unfamiliar dog during their first encounter.

Another major convergence of the different components of interactions with assistance dogs concerns the vocalizations produced by both groups. All children used vocalizations to communicate with the unfamiliar dog, a strategy also used by some children interacting with unfamiliar cats (6–10 years old; Mertens & Turner, 1988) or an unfamiliar guinea pig (6–12 years old; Grandgeorge et al., 2015). Our study provided a more detailed analysis by considering the function of these vocalizations. It revealed that both ASD children and TD children primarily used vocalizations to encourage or neutrally engage in interaction more than to interrupt it. This suggests that both groups were motivated to interact with the assistance dog. Furthermore, when TD children addressed their parents, their conversations were more frequently centered on the assistance dog than on other topics. Our research illustrated the social catalyst role of animals (McNicholas & Collis, 2000), which provides opportunities for social interactions between humans, and in this case, between children and their parents. This phenomenon of motivation to interact with a dog also applies to ASD children, as observed in our study through vocalizations, but also through other communication channels in different contexts (e.g., visually and physically, with an unfamiliar dog; Prothmann et al., 2009).

### 7.2. Convergences in the Identification Performance of Human and Canine Facial Expressions

The similarities between the two groups of children also extend to their ability to identify human and canine facial expressions (i.e., similar performance levels). Overall, both ASD children and TD children were better at recognizing human facial expressions than those of dogs. This result aligns with the idea that humans are specialized in recognizing expressions from their own species. For instance, TD children recognize human emotions more accurately than those of dogs, with their abilities improving with age and experience (Eretová et al., 2020; Correia-Caeiro et al., 2022). However, humans often struggle to interpret facial signals from other species, as emotional signals have evolved primarily for intra-specific communication rather than inter-specific interactions (Hawkins et al., 2021). This distinction appears early in development, as the attraction to faces, with a specialization for processing human faces, emerges between 6 and 9 months (Pascalis et al., 2002).

However, this result for ASD children is surprising as it contradicts existing literature. Indeed, most studies agree that ASD children have difficulties identifying human facial expressions due to their atypical face exploration patterns (e.g., Bölte & Poustka, 2003; Harms et al., 2010). We cannot rule out the possibility that the specific cohort in this research (i.e., ASD children with no severe symptoms) influenced the results, as well as the experimental protocol. Since there was no time constraint, the children may have felt less stress during the task. Additionally, the facial expressions used in the photographs may have been particularly explicit, making them easier to recognize given the five provided labels. These factors may have contributed to the observed results, even if similar findings have been reported by other authors. Indeed, Toutain et al. (2025) used the same identification test with young individuals with various disorders, including those with ASD, and found that they performed better at recognizing human facial expressions compared to canine facial expressions during their first test. However, their overall performance (including the recognition of human, canine, and feline expressions—the latter not being included in our study) was lower than that of the control group (without disorders). Interestingly, this difference disappeared after one year of regular exposure to an assistance dog in their classroom, suggesting a potential impact of prolonged exposure to dogs on their ability to interpret canine facial expressions.

Our study highlighted better recognition of anger in dogs than of other facial expressions. Anger in dogs is particularly recognizable due to distinctive patterns, such as visible teeth and specific mouth positions (open or closed) (Siniscalchi et al., 2018). In contrast, positive expressions in dogs can be harder to perceive, as they lack human-like indicators of joy, such as Duchenne smiles (Girard et al., 2019). However, 5–6-year-old TD children can recognize accurately certain canine facial expressions, such as anger and joy, regardless of their prior experience with dogs (Amici et al., 2019). These differences could be explained by breed-related effects, as our study used images of Golden Retrievers, Malinois, and mixed-breed dogs, whereas Amici et al. (2019) focused on “wolf-like” breeds such as German Shepherds and Huskies. Another finding of this study is that fear and sadness were the most difficult facial expressions for participants to identify in dogs, a result also reported in a recent study by Toutain et al. (2025). Specifically, this study revealed confusion between the two groups (TD and children with TND) when distinguishing between neutral and sad expressions on the same dog stimuli. This may be due to the subtler nature of facial expressions of fear and sadness in dogs, compared to anger or joy, which makes them harder to recognize based solely on facial cues. Children may rely more on a combination of behaviors to identify fear and sadness. Supporting this idea, Lakestani et al. (2014) found that children aged 6, 8, and 10 were more successful in interpreting dogs’ emotions in defensive-aggressive contexts, achieving a high accuracy rate of 92%. However, they struggled more with recognizing fear, with an accuracy rate of only 41%. In contrast, they showed moderate success in identifying emotions in friendly conditions, with an accuracy rate of 57%.

### 7.3. Similarities and Differences in Face Exploration (Experimenter vs. Assistance Dog)

We revealed that, when observing the experimenter and the assistance dog with the eye-tracking device, TD children spent more time looking at the dog’s face than at the experimenter’s face or the rest of the scene regardless of the experimental phase or instructions. These observations aligned with previous studies showing that dogs are visually attractive stimuli (images: Celani, 2002; real dogs: Dollion et al., 2021).

At a finer level, an assistance dog’s eyes were the part of the face most frequently observed by both ASD children and TD children, especially when asked about the dog’s emotional state or their perception of the dog. These findings corroborate previous studies conducted with both TD and ASD children using photos of animals, which reported that an animal’s eyes are the most visually engaging region of the face (dog: Muszkat et al., 2015; Duarte-Gan et al., 2023; dog, cat: Borgi et al., 2014; dog, horse, cat: Grandgeorge et al., 2016; dog, cat, horse, cow: Valiyamattam et al., 2020). Moreover, our research showed that TD children demonstrated the ability to adjust their visual attention to gather specific information based on questions about the dog’s perception and emotional state, relying more on their eyes during their reasoning. They were also able to apply this strategy to human faces, spending different amounts of time on each facial area depending on the context. This allowed them to adjust their information intake according to instructions (e.g., spend less time looking at the experimenter’s face during questions about the dog). This strategy is particularly efficient for dogs, as their eye region is crucial for recognizing their expressions (Siniscalchi et al., 2018). Furthermore, while the lower part of a dog’s face is generally less observed than their eyes, it is particularly important for assessing potentially threatening facial expressions (Siniscalchi et al., 2018).

### 7.4. Do Facial Exploration Patterns Modulate Facial Expression Identification Performance?

Our eye-tracking observations of facial exploration patterns in real-life conditions clearly correlate with performance in facial expression identification tasks, both for ASD children and TD children. Indeed, the more children, regardless of their diagnosis, focused their attention on a dog’s eye region, the better they identified the dog’s facial expressions. This is especially crucial for ASD children, as increased attention to a dog’s eyes during the eye-tracking phase was also associated with better results for the identification of human expressions. This suggests that children who can modulate their gaze, particularly by directing it toward the eyes, may find it easier to identify the facial expressions of both dogs and humans.

However, surprisingly, this link between preferential exploration of the experimenter’s eye region and human facial expression identification was evidenced, neither for ASD children nor for TD children. Additionally, and logically, ASD children who spent more time looking at the dog’s muzzle during eye-tracking recordings performed worse in facial expression identification (both for dogs and overall). This strategy may not be the most efficient, as although the lower part of a dog’s face conveys essential information in threat contexts, eyes—especially of breeds with highly mobile eye muscles, such as those in our study—provide rich cues in other contexts (Siniscalchi et al., 2018). With domestication, dogs have developed facial muscles around the eyes (absent in wolves), increasing their expressiveness for humans (Kaminski et al., 2019).

Moreover, ASD children who paid more attention to elements other than faces during the eye-tracking phases also had the lowest performance in facial expression identification for all presented stimuli. This observation aligned with the idea that reduced time spent looking at a face diminishes the ability to extract relevant information, such as movements or facial expressions (Morrison & Bellack, 1981). A reciprocal trend was also observed in TD children, where greater attention to a dog’s head during eye-tracking measurements was positively correlated with better performance in human facial expression identification tasks and overall results.

### 7.5. Do Facial Exploration Patterns Modulate Spontaneous Interaction with an Assistance Dog?

This ability to modulate attention was also reflected in spontaneous interactions with an assistance dog. Increased attention of ASD children to the dog’s head during eye-tracking measurements was associated with greater engagement in spontaneous interactions with a dog (i.e., more frequent gazes at the dog, increased physical contact, and greater interest in an intelligence-based toy). Similarly, the more TD children observed the dog’s head during the eye-tracking phase, the more they engaged in tug-of-war play. Conversely, when ASD children spent more time looking at elements other than faces during the eye-tracking phase, they engaged less in interaction (i.e., fewer gazes and fewer contacts with the dog). This link between visual attention and interaction is also supported by Dollion et al. (2021) who reported that ASD children who paid more attention to human and dog heads during real-life encounters (measured via eye-tracking) were engaged more in subsequent interactions with a dog.

Our study highlighted all children’s less interest in a dog’s ears during the eye-tracking phase, consistent with observations based on 2D stimuli (i.e., photographs; Grandgeorge et al., 2016; Valiyamattam et al., 2020). However, in real-life encounters, ears represent an information area on a dog’s head, particularly for communicating emotions (Siniscalchi et al., 2018). It would be interesting to further investigate this aspect in different contexts, such as in freer interactions between children and dogs.

### 7.6. Limits and Further Research

This study presents several limitations. First, the relatively small sample size limits the generalizability of the results and may have reduced the statistical power to detect significant effects. For the Chi-square tests, given the small sample size, we do not exclude the possibility of cumulative effects between specific pairs. Similarly, the lack of significant findings regarding factors influencing facial expression identification may be attributable to methodological limitations, such as a small sample size or insufficient statistical power to detect potential effects. A larger sample size could provide more robust evidence, and reveal effects that were not detectable in the current study. Second, it is important to note that the sex ratio in the sample is unbalanced, with a higher number of boys in the ASD group. This reflects the commonly observed prevalence of autism, which is more frequently diagnosed in boys. In contrast, the TD group is predominantly composed of girls, which may influence certain comparisons of results between the two groups. Thirdly, in Study 1 with eye-tracking measurements, particularly during fixation on the experimenter, the experimenter’s speech may have redirected the children’s attention towards her mouth rather than another facial feature. Nevertheless, the strength of this study lies in its ability to replicate conditions as close to real-life situations as possible. In everyday social interactions, it is common for one interlocutor to speak. It would be interesting to replicate this study using pre-recorded instructions delivered via a speaker rather than directly by the experimenter, to measure the potential impact of this variable.

Future research should investigate these links in interactions between children and their own pets (i.e., during daily interactions at home) using a longitudinal design to assess the impact of repeated exposure to dogs. Additionally, incorporating an interaction phase with a human partner (e.g., free interaction with another child) would help highlight the specificities of human-animal interactions and determine whether there is a connection between interactive behaviors, facial exploration strategies, and the ability to recognize others’ facial expressions. Finally, future studies should consider and control for the potential influence of having pets on gaze behavior, facial recognition abilities, and interactions with the assistance dog, as this could not be explored in the present study—nearly all participants had at least one pet in their household.

## 8. Conclusions

To conclude, this research is particularly innovative because it combines different levels of social information processing from information intake (visual attention) during real interaction to the understanding of social cues (facial expression recognition) and finally to the behavioral responses in a real-life interaction. Notably, our findings reveal a clear link between facial exploration strategies, understanding others (through facial expression identification), and behavioral expressions during interactions. Overall, the results from the three sub-studies revealed few differences between ASD children and TD children, except for some variations in visual attention and a more pronounced intra-group variability within the TD group than in the ASD group. To conclude, our research confirms the conclusions of a recent scoping review (Toutain et al., 2024) highlighting the fact that the visual attention of ASD individuals to animals differs from their visual attention to humans, being closer to that displayed by TD individuals. This particular visual attention may be one of the underlying mechanisms explaining the various benefits of human-animal interactions for people with ASD.

From an applied perspective, our results may help inform animal-assisted interventions by involving the animal as a support to draw analogies with humans, with dogs potentially serving as mediators in social contexts by helping children maintain their attention on a reassuring “social third party,” thereby facilitating interactions with others (adults or peers). For example, identifying parts of the dog’s face—often more attractive and engaging for children—can serve as a starting point to transfer attention to corresponding human facial features. However, it is essential to accompany these interventions with appropriate explanations, as simply directing attention to the correct area of the face does not in itself guarantee an accurate interpretation of the conveyed social or emotional information.

## Figures and Tables

**Table 1:** Characteristics of the study subjects (TD: typical development, ASD: Autism Spectrum Disorder; NA: not available). *: At least one animal is present in the household at the time of the study. Social Communication Questionnaire (SCQ): a score above 15 indicates a probable ASD diagnosis. Dunn’s Sensory Profile: a score between 38 and 122 indicates a significant sensory sensitivity difference compared to a group of TD children; a score between 123 and 149 indicates a probable sensory sensitivity difference compared to the same group; a score between 150 and 190 represents typical performance.

Participants Number	Group	Sex	Age (yo)	SCQ Score	Dunn Score	Presence of an Animal at Home *	Vision’s Particularities	Studies in Which the Participant Took Part
1	ASD	M	11	29	127	YES	NO	1, 2, 3
2	ASD	M	11	21	114	YES	*NA*	1, 2, 3
3	ASD	M	13	23	111	YES	NO	1, 2, 3
4	ASD	M	9	19	123	YES	*NA*	1, 2, 3
5	ASD	M	8	28	120	YES	NO	2, 3
6	ASD	M	16	22	*NA*	YES	Myopia	1, 2, 3
7	ASD	M	6	31	89	YES	Hyperopic	1, 2, 3
8	TD	M	8	0	180	YES	NO	1, 2, 3
9	TD	F	11	1	179	YES	Myopia	1, 2, 3
10	TD	F	13	4	190	YES	NO	1, 2, 3
11	TD	F	6	8	117	YES	Strabismus	2, 3
12	TD	F	11	0	178	YES	*NA*	1, 2, 3
13	TD	M	9	3	156	NO	NO	1, 2, 3
14	TD	F	9	1	162	YES	*NA*	1, 2, 3
15	TD	F	8	1	155	YES	Hyperopic	2, 3
16	TD	F	12	1	162	YES	NO	1, 2, 3
17	TD	F	9	1	158	YES	NO	1, 2, 3
18	TD	F	7	5	169	YES	NO	1, 2, 3
19	TD	F	11	0	182	YES	NO	1, 2, 3
20	TD	F	7	0	141	YES	*NA*	1, 2, 3

**Table 2:** Definitions of Areas of Interest (AOIs), description of their application and pictures for example.

**AOI Details**	**Experimenter**	**Assistance Dog**
	**Head** (circle with a maximum tolerance margin of half a hand around the head)	**Head** (circle around the head with a maximum tolerance margin of a quarter of a paw)
	**Eyes** (oval including both eyes when the head is facing forward, limited to the base of the nostrils)	**Eyes** (oval including both eyes when the head is facing forward, and only one eye when the head is in profile, as soon as the second eye is no longer visible)
	**Nose-mouth** (circle including the base of the nostrils, the mouth, and the base of the chin)	**Muzzle** (circle including the entire muzzle)
	**Ears** (not included due to technical challenge, small size, and limited visibility)	**Ears** (custom shape including the ear up to its lower insertion)
	**Other** (included the entire scene except the AOIs Head, Eyes, and Lower face)	**Other** (included the entire scene except the AOIs Head, Eyes, Lower face, and Ears)
**Example**	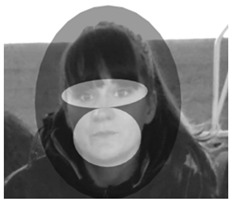	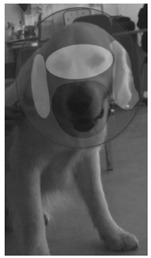

**Table 3:** Behavioural repertoire.

Behavioural Category	Definition	Subtypes and Behaviours
Distance between child and assistance dog	Distance between the area of the child’s body closest to the assistance dog.	Direct contact, <1 arm, 1 to 2 arms, >2 arms, further away.
Distance between child and parent	Distance between the area of the child’s body closest to the parent.	Direct contact, <1 arm, 1 to 2 arms, >2 arms, further away.
Play behaviours	The child engages spontaneously with a dog in a playful activity.	Types of games: toy veterinary kit, rope, intelligence toy, chase, other (e.g., hold on to the lead, child lies down and strokes the dog while laughing, child throws another object at the assistance dog).
Gaze orientation of child	Global orientation of head and gaze.	Targets: assistance dog, parent, assistance dog-related object, self-centred, environment, undetermined.
Distance modulation with the assistance dog	The child approaches or backs away from the dog with both feet.	Types: approaching the assistance dog, backing away from an assistance dog.
Child’s contact with assistance dog	The child had a part of her/his body in physical contact with assistance dog’s body.	Type of contact: active (voluntary, involves movement) or passive (involuntary, does not involve movement).
Care behaviour by child	The child’s behaviour to care for the assistance dog or meet its needs.	The child brushes the assistance dog, gives it water or a kibble.
Vocalisation of child directed to assistance dog	The child used some vocalisation directed to assistance dog	Type of verbalisation: encourages interaction, neutral, stops interaction.
Vocalisation of child directed to parent	The child used some vocalisation directed to parent.	Nature of comments: about the assistance dog, other.
Other type of vocalisations	The child gives non-vocal vocal cues when interacting with the assistance dog.	Types: onomatopoeia (sounds, cries, noises to call the dog), laughter, smiles.
Joint attention	The child shares a moment of visual attention with the dog after following its gaze.	NA
Self-centred behaviour	The child’s behaviour or action is self-centred.	NA

NA: not available.

**Table 4:** Times (mean percentage) spent at different distances from an assistance dog and their parent. Friedman test. *p* > 0.05.

Distance from	Assistance Dog		Parent	
	ASD	TD	ASD	TD
	Mean ± SD (%)	Mean ± SD (%)	Mean ± SD (%)	Mean ± SD (%)
Direct contact	32.78 ± 21.00	34.46 ± 23.56	0.45 ± 0.89	0.25 ± 0.51
<1 arm	37.93 ± 8.71	44.80 ± 16.93	4.55 ± 9.43	0.68 ± 1.37
1 to 2 arms	11.88 ± 10.12	12.15 ± 7.51	2.28 ± 2.40	9.43 ± 11.61
>2 arms	17.17 ± 14.07	8.59 ± 5.48	92.72 ± 11.25	89.64 ± 11.92
**Statistics**	X^2^ = 19.8; *p* < 0.001	X^2^ = 42.9; *p* < 0.001	X^2^ = 22.8; *p* < 0.001	X^2^ = 43.8; *p* < 0.001

**Table 5:** Times (mean percentage) spent performing different types of play for ASD children and TD groups. Friedman test: *p* < 0.05.

	ASD	TD
	Mean ± SD (%)	Mean ± SD (%)
Other play	5.41 ± 8.22	7.77 ± 11.38
Toy veterinary kit	12.83 ± 14.69	4.95 ± 5.91
Rope play	19.94 ± 22.04	13.21 ± 9.98
Running with the dog	2.44 ± 6.46	0.62 ± 1.53
Intelligent toy	4.22 ± 5.75	5.98 ± 4.57
Not playing	55.15 ± 24.99	67.46 ± 14.49
**Statistics**	X^2^ = 17.7; df = 5; *p* = 0.003	X^2^ = 37; df = 5; *p* < 0.001

**Table 6:** Visual attention (mean percentage) for both groups. Friedman test, *p* < 0.05.

	ASD	TD
	Mean ± SD (%)	Mean ± SD (%)
Assistance dog	51.13 ± 16.94	66.59 ± 10.15
Assistance dog’s objects	22.30 ± 10.18	16.42 ± 8.25
Environment	13.29 ± 8.59	8.99 ± 4.21
Parent	6.20 ± 6.74	5.20 ± 2.65
Self-centered	1.99 ± 1.84	0.85 ± 0.60
Indeterminate	5.09 ± 5.40	1.95 ± 2.44
**Statistics**	X^2^ = 26; df = 5; *p* = 0.003	X^2^ = 54.7; df = 5; *p* < 0.001

**Table 7:** Time spent (mean duration and average percentage) engaging in relation to contact type with a dog by ASD children and TD children. Wilcoxon test: *p* < 0.05.

	ASD	TD
	Mean ± SD Duration (s)	Mean ± SD Duration (s)
Active contact (duration)	57.95 ± 49.12	137.98 ± 116.61
Passive contact (duration)	109.21 ± 82.60	52.78 ± 41.34
**Statistics**	W = 5, *p* = 0.30	W = 82; *p* = 0.008
	**Mean ± SD (%)**	**Mean ± SD (%)**
Active contact (occurrences)	11.15±	16.04 ± 6.44
Passive contact (occurrences)	15.97±	9.24 ± 4.76
**Statistics**	W = 7, *p* = 0.3	W = 85, *p* = 0.007

**Table 8:** Vocalizations (mean percentage) directed towards dogs or parents by ASD children and TD children. Friedmann Test and Wilcoxon test: *p* < 0.05.

	ASD	TD
	Mean ± SD (%)	Mean ± SD (%)
Encourage interaction with the assistance dog	14.89 ± 9.94	21.98 ± 11.22
Stop interaction with the assistance dog	1.37 ± 3.20	0.07 ± 0.26
Neutral verbalization towards the assistance dog	18.78 ± 17.14	16.82 ± 7.51
**Statistics** (Friedman test)	X^2^ = 9.55, *p* = 0.008	X^2^ = 19.86, *p* < 0.001
Talks about the assistance dog with the parent	7.21 ± 10.69	9.40 ± 5.71
Talks about something other than the assistance dog with the parent	6.36 ± 7.72	5.28 ± 3.74
**Statistics** (Wilcoxon test)	W = 11, *p* = 0.690	W = 68, *p* = 0.025

**Figure A1:**
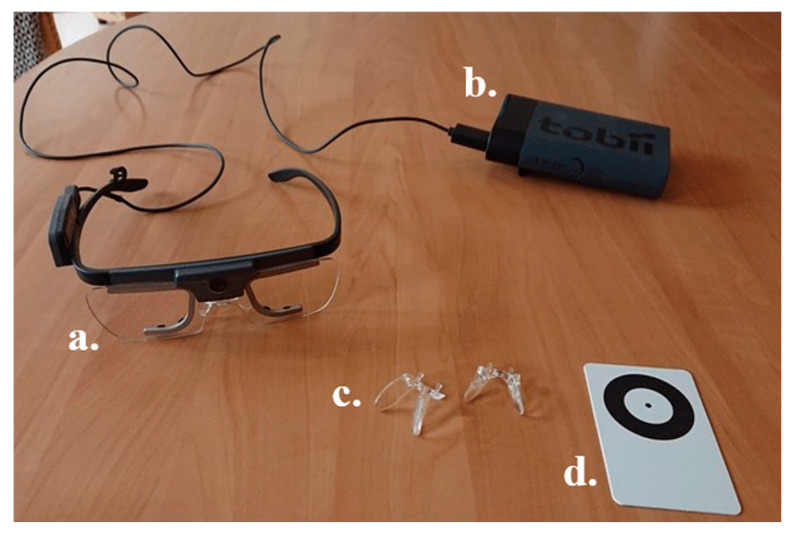
**Eye-tracking glasses system—Tobii Glasses Pro 2** a. **Glasses** b. **Power unit**: A power box that supplies energy to the glasses, connected to the computer via Wi-Fi, enabling more freedom of movement while continuously recording eye data. c. **Nose pads in various sizes**: Adjustable nose pads that allow for a comfortable fit, especially for younger children, ensuring better accuracy and comfort during long sessions. d. **1-point calibration card**: A tool used to calibrate the eye-tracking system at the start of the experiment, ensuring precise tracking based on the individual’s eye movements and position.

## Data Availability

Data will be available upon the author’s request.

## Notes

1 A shorter measurement time than the total recording duration (20 s) was due to temporary loss of eye-tracking signal caused by participants’ eye movements (e.g., looking too high or too low relative to the glasses’ sensors, resulting in data loss). This presentation phase generated greater attentional shifts between the experimenter and an assistance dog, creating more signal interferences for some individuals.

2 The term “emotion” was chosen over “facial expression” because it is better understood by children of the age group included in this study, especially by ASD children.

## Abbreviations

The following abbreviations are used in this manuscript:

ASD	Autism Spectrum Disorder
TD	Typical Development

## Appendix B

**Table A1:** Mean percentage of eye-tracking measurements by phase (in seconds).

Variables		ASD		TD	
		Mean ± SD (%)	SD	Mean (%)	SD
**Assistance dog presentation**	Other	17.32 ± 23.00	23.00	20.22 ± 9.12	9.12
	Dog’s head	58.32 ± 21.75	21.75	52.07 ± 18.13	18.13
	Muzzle	14.92 ± 10.89	10.89	15.43 ± 8.34	8.34
	Dog’s eyes	23.86 ± 22.44	22.44	25.33 ± 20.45	20.45
	Experimenter’s head	24.36 ± 24.81	24.81	27.71 ± 18.22	18.22
	Dog’s ears	2.75 ± 5.77	5.77	2.55 ± 3.06	3.06
	Nose-mouth	8.33 ± 11.01	11.01	12.35 ± 12.19	12.19
	Experimenter’s eyes	9.84 ± 10.89	10.89	8.24 ± 7.63	7.63
**Questions about assistance dog**	Other	18.01 ± 16.09	16.09	18.01 ± 13.29	13.29
	Dog’s head	70.80 ± 17.75	17.75	72.28 ± 13.24	13.24
	Muzzle	20.71 ± 17.50	17.50	15.90 ± 10.38	10.38
	Dog’s eyes	29.49 ± 28.94	28.94	22.07 ± 11.83	11.83
	Experimenter’s head	11.19 ± 8.62	8.62	9.71 ± 7.77	7.77
	Dog’s ears	2.71 ± 1.85	1.85	9.16 ± 8.04	8.04
	Nose-mouth	3.62 ± 5.92	5.92	3.72 ± 5.04	5.04
	Experimenter’s eyes	3.88 ± 5.32	5.32	3.17 ± 3.09	3.09
**Gaze fixation on the experimenter**	Other	14.00 ± 17.78	17.78	0.22 ± 0.69	0.69
	Experimenter’s head	86.00 ± 17.78	17.78	99.78 ± 0.69	0.69
	Nose-mouth	24.85 ± 30.65	30.65	34.62 ± 40.42	40.42
	Experimenter’s eyes	42.99 ± 39.60	39.60	47.77 ± 39.31	39.31
**Gaze fixation on the assistance dog**	Other	20.05 ± 24.81	24.81	2.28 ± 7.11	7.11
	Dog’s head	79.95 ± 24.81	24.81	97.72 ± 7.11	7.11
	Muzzle	12.14 ± 19.40	19.40	14.53 ± 25.85	25.85
	Dog’s eyes	50.43 ± 43.14	43.14	60.80 ± 28.28	28.28
	Dog’s ears	3.27 ± 3.91	3.91	4.77 ± 13.72	13.72
